# Knowledge mapping and bibliometric analysis of medical knee magnetic resonance imaging for knee osteoarthritis (2004–2023)

**DOI:** 10.3389/fsurg.2024.1387351

**Published:** 2024-09-11

**Authors:** Juntao Chen, Hui Xu, Hang Zhou, Zheng Wang, Wanyu Li, Juan Guo, Yunfeng Zhou

**Affiliations:** ^1^ College of Acupuncture and Tuina, Henan University of Chinese Medicine, Zhengzhou, China; ^2^ Tuina Department, The Third Affiliated Hospital of Henan University of Chinese Medicine, Zhengzhou, China

**Keywords:** bibliometric, knee osteoarthritis, magnetic resonance imaging, knowledge graph, CiteSpace, VOSviewer

## Abstract

**Objectives:**

Magnetic resonance imaging (MRI) is increasingly used to detect knee osteoarthritis (KOA). In this study, we aimed to systematically examine the global research status on the application of medical knee MRI in the treatment of KOA, analyze research hotspots, explore future trends, and present results in the form of a knowledge graph.

**Methods:**

The Web of Science core database was searched for studies on medical knee MRI scans in patients with KOA between 2004 and 2023. CiteSpace, SCImago Graphica, and VOSviewer were used for the country, institution, journal, author, reference, and keyword analyses.

**Results:**

A total of 2,904 articles were included. The United States and Europe are leading countries. Boston University is the main institution. Osteoarthritis and cartilage is the main magazine. The most frequently cocited article was “Radiological assessment of osteoarthrosis”. Guermazi A was the author with the highest number of publications and total references. The keywords most closely linked to MRI and KOA were “cartilage”, “pain”, and “injury”.

**Conclusions:**

The application of medical knee MRI in KOA can be divided into the following parts: (1). MRI was used to assess the relationship between the characteristics of local tissue damage and pathological changes and clinical symptoms. (2).The risk factors of KOA were analyzed by MRI to determine the early diagnosis of KOA. (3). MRI was used to evaluate the efficacy of multiple interventions for KOA tissue damage (e.g., cartilage defects, bone marrow edema, bone marrow microfracture, and subchondral bone remodeling). Artificial intelligence, particularly deep learning, has become the focus of research on MRI applications for KOA.

## 1 Introduction

Knee osteoarthritis (KOA) is a degenerative disease characterized by clinical symptoms and joint tissue deformations. KOA is associated with damage to the articular cartilage and related pain, swelling, and joint stiffness, which are the main causes of disability (1). As a chronic degenerative disease, the pathological damage caused by KOA involves inflammation, injury, genetics, and other factors (2–4). Due to the depth of research, various treatment methods, including nondrugs, drugs, and surgery, play certain advantages in the clinical treatment of KOA. However, owing to the complexity of its pathogenesis, there is currently no single treatment for the complete eradication of KOA (5–8). The incidence of KOA is high and is expected to increase (9). Studies have shown that the overall prevalence of hip and knee OA worldwide is approximately 300 million (10). The prevalence of mild, moderate, and severe KOA in China is 1.5%, 3.3%, and 3.9%, respectively, and the incidence in women is much higher than that in men (11). According to a study by American scholars, about 9.29% of people over the age of 60 in the United States have been diagnosed with symptomatic KOA (12). Diagnosis of KOA is critical given its high incidence. Imaging plays an important role in KOA diagnosis.

The imaging methods used to detect KOA include ultrasonography, radiography, computed tomography, and magnetic resonance imaging (MRI), all of which have advantages (13, 14). Tissue damage, including cartilage and meniscal damage, can affect the onset of KOA. However, MRI is more sensitive to changes such as cartilage injury, soft tissue lesions, and chondroedema than radiographs, which can only evaluate the knee joint space. This helps to assess the occurrence of KOA early and prevent it as soon as possible (15). The value for the diagnosis and treatment of KOA has gained increasing attention. For example, an imaging omics-clinical nomogram model based on MRI-bone marrow edema showed good performance in the diagnosis of early osteoarthritis (16). Meanwhile, using semi-quantitative methods, MRI can classify KOA into meniscus, cartilage, inflammation, and other structural phenotypes. The Whole Organ Magnetic Resonance Imaging Score (WORMS) and other MRI-based scoring systems have also been developed for epidemiological studies and risk factor analysis of KOA (17, 18). Quantitative morphological cartilage assessment is occasionally performed using MRI, including the segmentation of bone, cartilage, bone surface, cartilage surface, and cartilage morphology measurements. Component MRI analysis and MRI pulse sequence analysis have also been applied in imaging research for KOA, and each has certain advantages (19–22). MRI provides a more detailed observation of cartilage and bone marrow edema; therefore, it can be used for the diagnosis and clinical treatment of KOA (23, 24). For example, in an imaging omics-clinical nomogram model based on MRI-bone marrow, edema showed good performance in the diagnosis of early osteoarthritis (16). Studies by Salikhov et al. have also shown that in patients with type 1–2 KOA cartilage defects, MRI can accurately assess cartilage damage and guide the treatment of stromal vascular parts. MRI is helpful for evaluating the repair effect of the Stromal vascular fraction (SVF) on cartilage injury and provides a clearer basis for clinical treatment (25). Due to the advantages and extensive application of medical knee MRI in the diagnosis, evaluation, and treatment of KOA, it is of practical significance to clarify the application status of MRI in KOA to analyze future research hotspots and guide further research. Bibliometrics provides the possibility of conducting research as a scientific method for literature analysis.

Bibliometrics is a popular and rigorous method for exploring and analyzing large amounts of scientific data. Bibliometrics enabled us to reveal the current research situation and differences in a specific field, as well as the emerging direction in this field (26). Bibliometrics analysis can use the information of the network literature database to quantitatively and qualitatively evaluate the main content and hot spots of a certain field (27). We employ bibliometrics to provide technical support for this study to deeply investigate the current global application of medical knee MRI in patients with KOA and analyze future research trends.

## 2 Materials and methods

### 2.1 Data source

On January 7, 2024, 6,050 articles were identified in the Web of Science (WoS) Core Collection: the Science Citation Index Expanded (SCIE) and the Social Science Citation Index (SSCI). The bibliometric analysis was based on WoS, which is considered the best bibliometric database.

### 2.2 Search strategy and data collection

The search strategies are summarized in Table 1. One author conducted a preliminary screening (J. C.) and then provided the results to the other two authors for rescreening (H. Z., Z. W.). Patients with serious knee diseases such as cancer, tuberculosis, hip osteoarthritis, acute knee injury, rheumatoid arthritis, gouty arthritis, psoriatic arthritis, and other non-KOA studies were excluded. A total of 2,996 articles remained for potential inclusion. CiteSpace (version 6.2. r7) was used to analyze literature. A total of 92 duplicate articles were excluded and 2,904 were ultimately included.

**Table 1:** Literature retrieval methods.

Set	Result	Search query
#1	27,314	(((((((TS = (Joint Magnetic Resonance Imaging)
		OR TS = (Knee Magnetic Resonance Imaging)
		OR TS = (Knee MRI)) OR TS = (Knee Joint MRI)) OR TS = (Knee Joint MR))
		OR TS = (MRI of Knee))
		OR TS = (Prayer Bones MRI))
		OR TS = (Prayer Bones MR)
#2	58,007	((((((((((TS = (Knees Osteoarthritis))
		OR TS = (Osteoarthritis, Knees))
		OR TS = (KOA)) OR TS = (Osteoarthritis of Knee))
		OR TS = (Osteoarthritis Of Knees)))
		OR TS = (Osteoarthritis of Articular Genu)))))
		OR TS = (Osteoarthritis of the Knee))
		OR TS = (Knee Osteoarthritis)
#3	6,050	Index = WoS Core Collection
		Editions = SCI-EXPANDED and SSCI
		Timespan = 2004–2023
		Language = English
		Document types = Article and review article
		#1 and #2
#4	2,996	After rescreening
#5	2,904	After removing duplicate literature using CiteSpace (6.2.r7)

### 2.3 Bibliometric analysis

CiteSpace and VOSviewer are the main data visualization tools used in bibliometric studies. They present the structure, laws, and distribution of knowledge on a certain subject in the form of scientific knowledge graphs to discover advances and research frontiers in specific fields. We used CiteSpace and VOSviewer to complete relevant analyses of countries, institutions, journals, authors, references, and keywords. ScimagoGraphica is a grammar-based digital imaging tool that was used to complete national geographic distribution, publication trends, references, and keyword network connection analyses.

## 3 Results

### 3.1 Annual number and trend of publications

**Figure 1:**
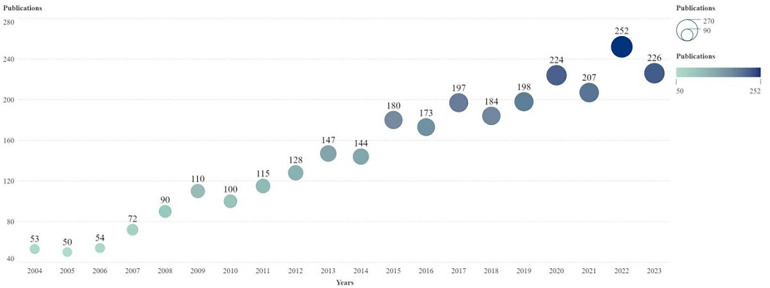
Annual publication trend chart of research on the application of MRI in patients with KOA. The horizontal axis is the year and the vertical axis is the number of published articles. The node size and color represent the number of publications. Smaller nodes and lighter colors represent fewer publications. The number of posts per year is indicated above the node. MRI, magnetic resonance imaging.

A literature review was conducted from January 1, 2004, to December 31, 2023. A total of 2,904 articles were published on the application of medical knee MRI in patients with KOA, with an average of 145 articles published annually. After 2009, the number of documents issued annually exceeded 100; since 2020, the number has exceeded 200. With an in-depth understanding of KOA pathogenesis and further development of MRI technology, the number of publications will also increase (Figure 1). According to the results, the total number of research studies and annual average number of medical knee MRI applications in KOA are increasing, which has certain research prospect.

### 3.2 National analysis

A national analysis revealed that from 2004 to 2023, 76 countries participated in MRI-related research on KOA. The United States had the largest number of publications (1,260), followed by Australia (618) and Germany (506). The United States had the highest intermediary centrality (0.35), followed by Germany (0.32), Australia, and the United Kingdom (0.21). The United States had the highest total citation frequency of 57,311, followed by Australia (24,608) and Germany (20,498) (Table 2).

**Table 2:** Top ten countries in the field of publications, total citation frequency, and mediation centrality in the application of MRI in KOA.

Rank	Publications	Countries	Citations	Countries	Centrality	Countries
1	1,260	USA	57,311	USA	0.35	USA
2	618	Australia	24,608	Australia	0.32	Germany
3	506	Germany	20,498	Germany	0.21	Australia
4	360	China	11,694	UK	0.21	UK
5	303	UK	8,278	Sweden	0.13	Italy
6	207	Canada	7,270	Canada	0.11	France
7	168	Netherlands	5,914	Netherlands	0.10	Spain
8	164	Japan	4,856	France	0.09	China
9	114	Switzerland	4,205	China	0.06	Switzerland
10	109	Sweden	3,776	Switzerland	0.05	Netherlands

**Figure 2:**
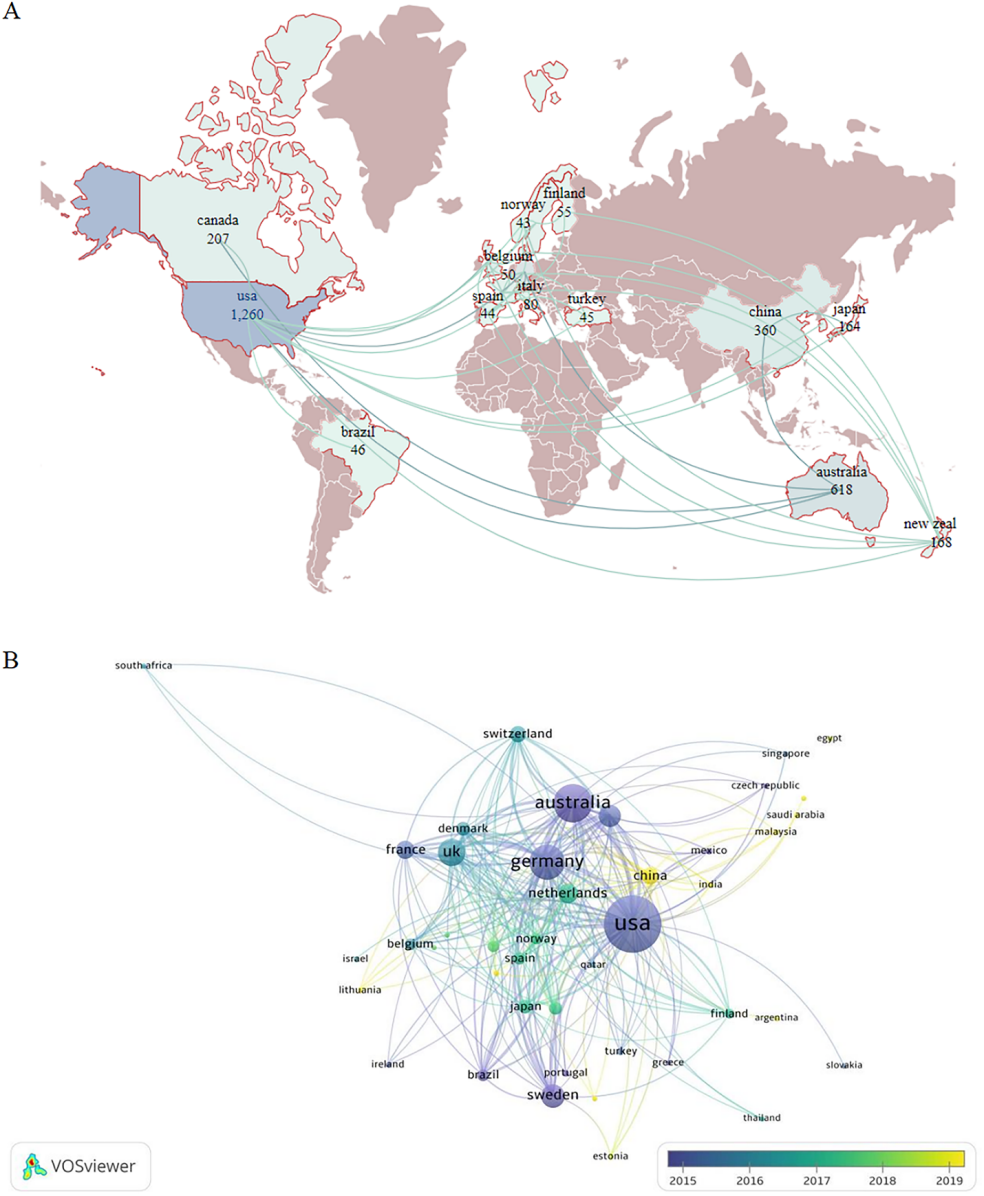
**(A)** Geographic map of the national document distribution. The figure shows countries with more than 40 publications. Dark brown color represents countries with fewer than 40 publications, whereas the light blue color represents those with a low number of publications. The more connections between the two countries in the picture, the closer the country is to other countries. The numbers below each country represent the number of publications in the field. **(B)** National network connectivity map. Larger node indicates more total literature citations in the country. The color of the node represents the publication time of the country. Darker color indicates earlier publishing time.

The total citation frequencies of the United States, Australia, and Germany were consistent with the rankings of their published papers, proving that these three countries have a strong influence in this field. The national geographic distribution map of countries shows that the United States and Australia have formed close ties with European countries such as Germany and France. China and Japan have numerous publications and are closely linked (Figure 2A). As can be seen from Figure 2B, the literature published in China, India, Saudi Arabia, and other countries is relatively recent, indicating that these countries are strengthening their research on the application of MRI in KOA. In general, the United States, Australia, and some European countries are key countries in this field, and both the number of publications and the total number of citations are among the top worldwide. Among Asian countries, China and Japan are the major publishing countries.

### 3.3 Institutional analysis

According to data analysis, 2,873 institutions have published literature on the application of medical knee MRI in KOA. We analyzed 2,873 institutions involved in MRI research on patients with KOA over the past 20 years, of which 151 institutions published >10 articles. Boston University ranked first, with 400 publications, followed by the University of California, San Francisco (314) and Monash University (213). The University of A Coruña INSERM had the highest intermediary centrality of 0.7, followed by the Leeds Biomedical Research Centre (0.67) and Feinberg School of Medicine (0.67). Boston University had the highest citation frequency of 22,308, followed by the University of California San Francisco (17,376) and Monash University (9,354) (Table 3).

**Table 3:** Top 10 institutions in the field of publications, total citation frequency, and mediation centrality.

Rank	Publications	Institutions	Citations	Institutions	Centrality	Institutions
1	400	Boston University	22,308	Boston University	0.7	University of A Coruña Inserm
2	314	University of California San Francisco	17,376	University of California, San Francisco	0.67	Leeds Biomedical Research Centre
3	213	Monash University	9,354	Monash University	0.67	Feinberg School of Medicine
4	146	University of Tasmania	6,406	Lund University	0.66	Lyon Civil Hospice
5	145	University of Sydney	5,706	Klinikum Augsburg	0.63	Institut National De La Sante Et De La Recherche Medicale
6	104	Langen University, Nuremberg	5,295	University of Sydney	0.63	University of Sydney
7	92	Paracelsus Medical School	4,877	Stanford University	0.62	Royal North Shore Hospital
8	84	Lund University	4,674	University of Lowa	0.61	Chapel Allerton Hospital
9	81	Stanford University	4,541	University of Tasmania	0.61	University of Sheffield
10	71	University of Lowa	3,929	University of Leeds	0.60	Beth Israel Deaconess Medical Center

**Figure 3:**
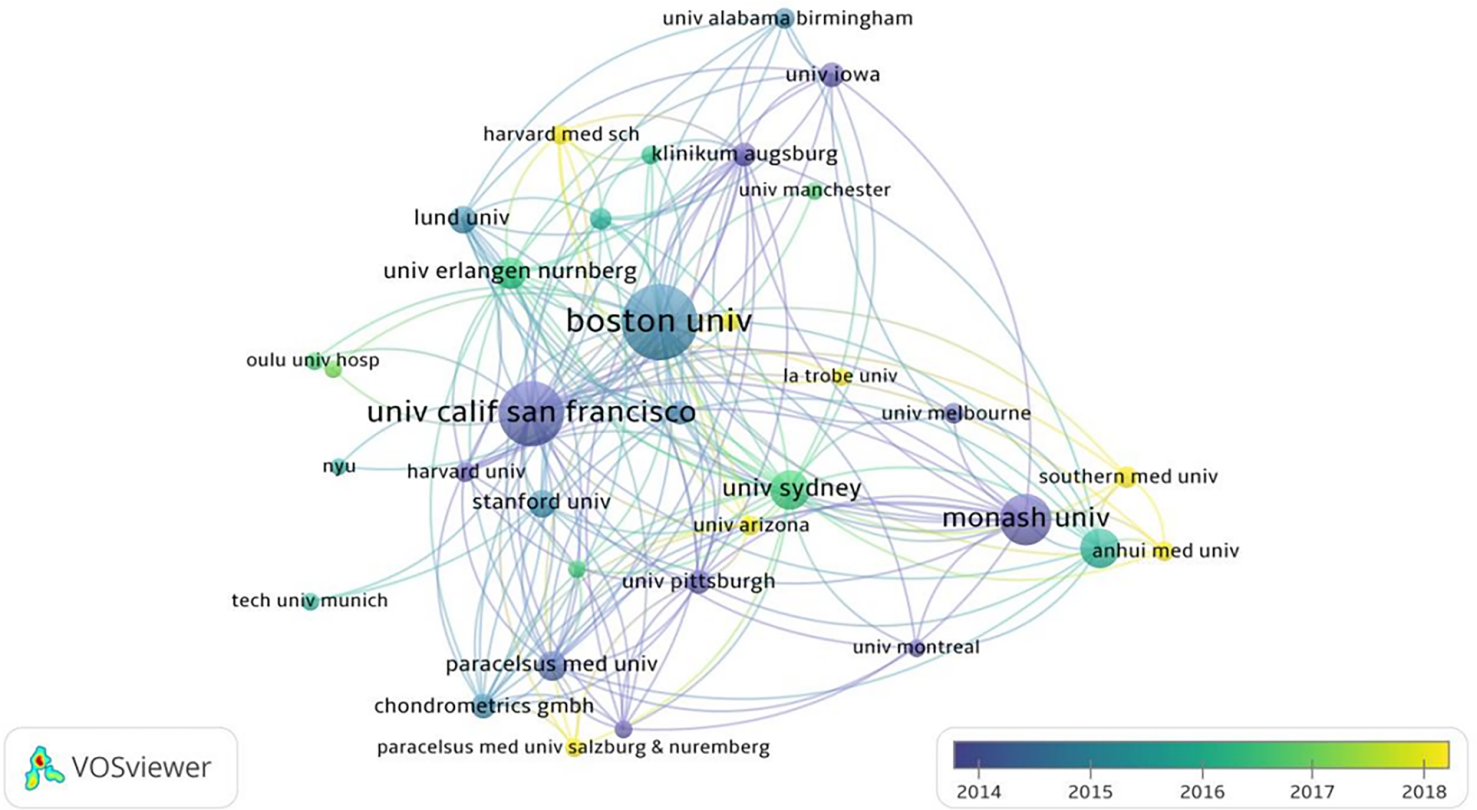
Institutional network connectivity diagram. The size of the nodes represents the number of posts by the agency. Larger nodes indicates more the number of posts. The color of the node represents the publishing time. Darker color indicates earlier the publishing time.

Boston University had the most citations, proving that it has considerable academic influence and research achievements in this field. In contrast, the University of A Coruña INSERM and the Leeds Biomedical Research Centre had the highest intermediary centrality, which may have been affected by the research content of the articles. The University of A Coruña INSERM has focused on the genetics of OA and the standardization of clinical symptoms and treatment. Research from the Leeds Biomedical Research Centre has focused on MRI studies of rheumatic immune system diseases and the manifestations of several knee diseases (28–30). Few studies in the literature are from the Feinberg School of Medicine, and most have focused on analyzing the risk factors for KOA (31, 32). As shown in Figure 3, institutions such as Southern Medical University and Anhui Medical University later faced issues regarding this topic. These institutions have invested considerable research into the application of mechanical knee MRI in KOA and have achieved certain results. In short, institutions in the United States and Europe have a large number of publications, and the content of different institutions in this field is inconsistent. In recent years, institutions in China have paid close attention to the content of this field and have published a certain number of posts.

### 3.4 Journal analysis

**Figure 4:**
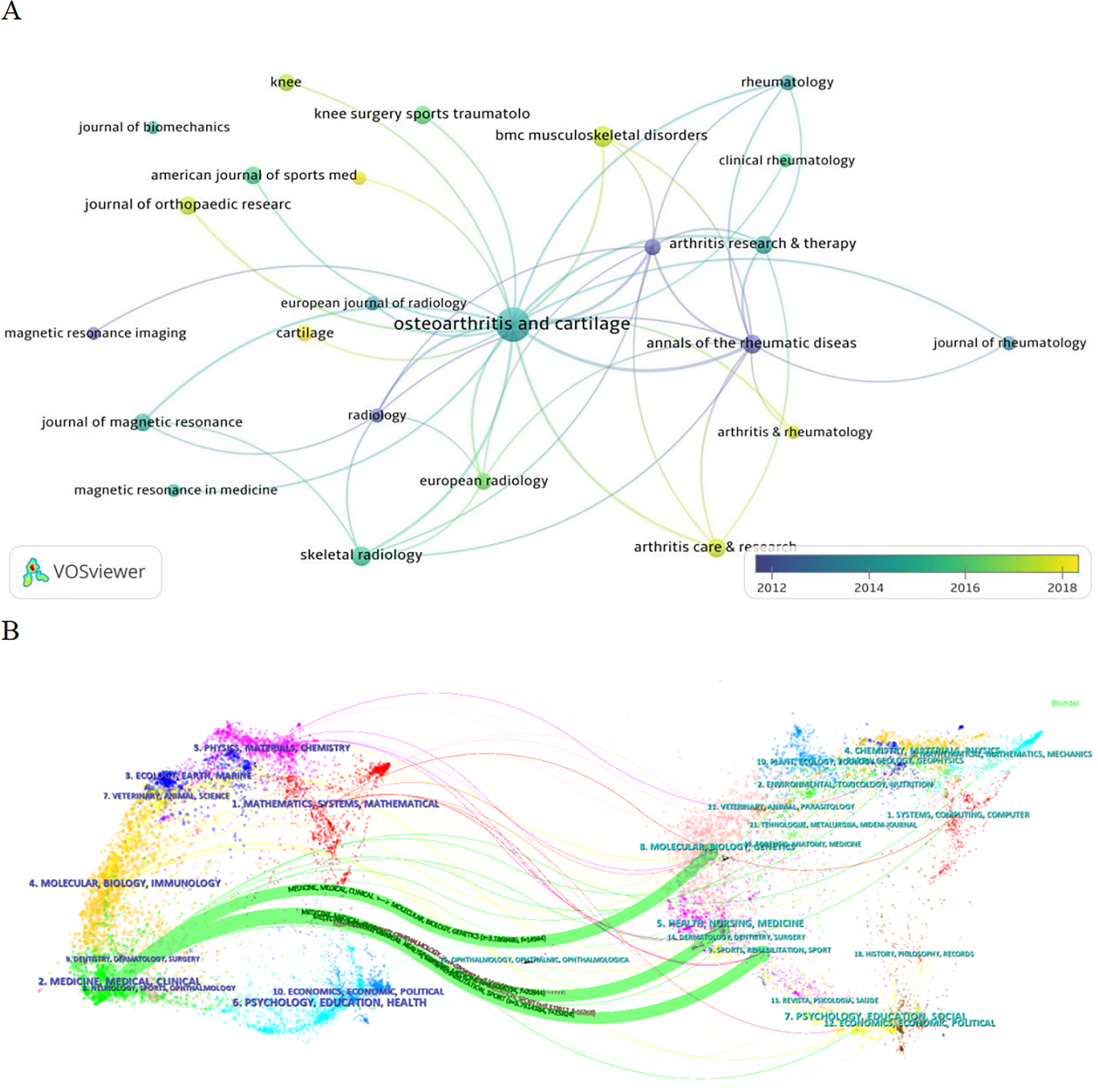
**(A)** Journal network connectivity map. The size of the nodes represents the number of articles published, where the larger the nodes, the more research articles the journal publishes in this field. The color of the node represents the time of the publication, where the darker the color, the earlier the magazine publishes the article. **(B)** Double image overlay of the journal. The left side is the cited journal, and the right side is the back cited journal. The colors of the connecting lines represent the citation journals derived from the same category. Thickness of the connection represents the number of references (i.e., as the number of references increases, so does the thickness of the connection).

A total of 470 journals published articles on the application of medical knee MRI in KOA, of which 29 published >20 articles. The journal with the greatest number of articles was Osteoarthritis and Cartilage (445), followed by BMC Musculoskeletal Disorders (97) and Skeletal Radiology (84). Osteoarthritis and Cartilage ranked first in the number of citations with 20,320, followed by Annals of the Rheumatic Diseases (7,494) and Arthritis and Rheumatism (6,411). Osteoarthritis and Cartilage had the greatest total citation frequency (16,155), followed by Annals of Rheumatic Diseases (7,697) and Arthritis and Rheumatism (7,380) (Table 4). Osteoarthritis and Cartilage had the highest number of publications and total citations and was a core journal in this field (Figure 4A).

**Table 4:** Top 10 journals in the field of publications, total citation frequency, and total citation frequency of application of MRI in KOA.

Rank	Publications	Journals	IF^a^	Citations	Journals	Co-citations	Journals	IF^a^
1	445	Osteoarthritis and Cartilage	7	20,320	Osteoarthritis and Cartilage	16,155	Osteoarthritis and Cartilage	7
2	97	BMC Musculoskeletal Disorders	2.3	7,494	Annals of the Rheumatic Diseases	7,697	Annals of the Rheumatic Diseases	27.4
3	84	Skeletal radiology	2.1	6,411	Arthritis and Rheumatism	7,380	Arthritis and Rheumatism	8.955
4	78	Annals of the Rheumatic Diseases	27.4	4,835	American Journal of Sports Medicine	3,890	Radiology	19.7
5	74	Arthritis Care and Research	4.7	3,662	Arthritis Research & Therapy	3,397	American Journal of Sports Medicine	4.8
6	72	Journal of Orthopaedic Research	2.8	3,079	Radiology	2,595	Journal of Rheumatology	3.9
7	70	Arthritis Research & Therapy	4.9	2,166	Knee Surgery Sports Traumatology Arthroscopy	2,005	Journal of Bone and Joint Surgery-American Volume	5.3
8	69	Knee Surgery Sports Traumatology Arthroscopy	3.8	1,960	Journal of Magnetic Resonance Imaging	1,979	Magnetic Resonance in Medicine	3.3
9	65	American Journal of Sports Medicine	4.8	1,829	Rheumatology	1,948	Arthritis Research & Therapy	4.9
10	59	Journal of Magnetic Resonance Imaging	4.4	1,660	Journal of Orthopaedic Research	1,946	Clinical Orthopaedics and Related Research	4.3

The IFs of arthritis and rheumatism are from the 2015 journal citation report. The remaining journal IFs are all from the 2022 journal citation reports.

^a^ IF, impact factor.

According to the results of the journal double-image overlay analysis, medical knee MRI and KOA studies were published in orthopedic journals, but were also relevant to molecular science, nursing, sports, rehabilitation, and other fields (Figure 4B). The results show that journals published in this field have a wide range and high quality, and articles published in orthopedic journals are more likely to receive the attention of scholars.

### 3.5 Author analysis

In total, 10,884 authors participated in research on the application of medical knee MRI in KOA. Nineteen authors have published more than 50 articles. Guermazi A was identified as the author with the largest number of publications (309), followed by Roemer FW (183), Eckstein F (148), and Hunter DJ (148). Guermazi (0.22) had the highest intermediary centrality, followed by Cicuttini (0.21) and Hunter (0.18). Guermazi also had the highest number of citations (16,680), followed by Hunter DJ (8,908) and Felson DT (8,882) (Table 5).

**Table 5:** The authors of the top 10 publications, total citation frequency, and mediation centrality in research on the application of MRI in KOA.

Rank	Publications	Authors	Citations	Authors	Centrality	Authors
1	309	Guermazi A	16,680	Guermazi A	0.22	Guermazi A
2	183	Roemer FW	8,908	Hunter DJ	0.21	Cicuttini FM
3	148	Eckstein F	8,882	Felson DT	0.18	Hunter DJ
4	148	Hunter DJ	8,472	Roemer FW	0.13	Eckstein F
5	124	Felson DT	6,591	Eckstein F	0.1	Link Thomas M
6	122	Cicuttini FM	5,955	Cicuttini FM	0.08	Lynch J
7	91	Link Thomas M	3,463	Nevitt Michael C	0.07	Changhai Ding
8	72	Changhai Ding	3,287	Link Thomas M	0.07	Nevitt Michael C
9	72	Jones Graeme	2,777	Wluka Anita	0.05	Wluka Anita
10	72	Nevitt Michael C	2,554	Majumdar Sharmila	0.05	Majumdar S

**Figure 5:**
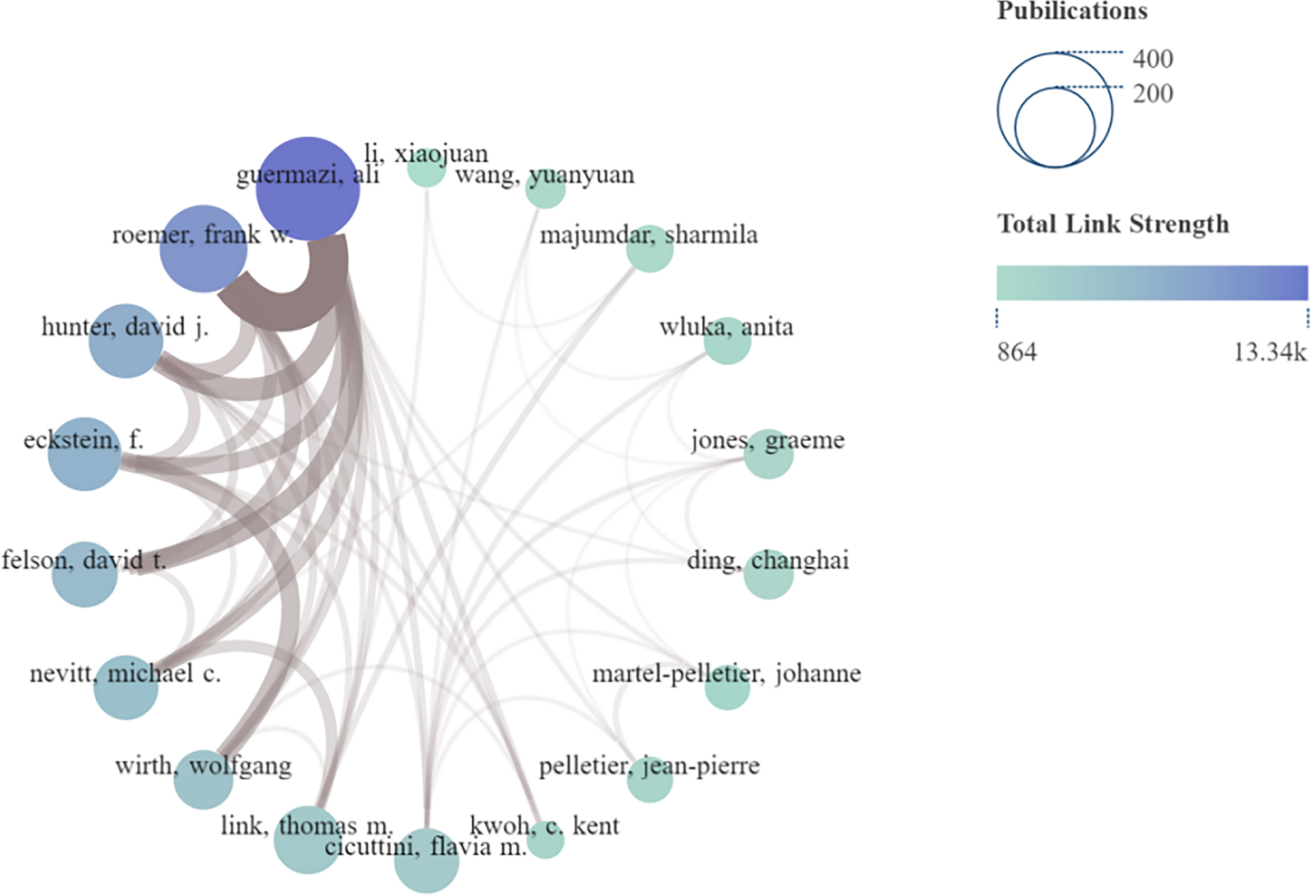
Network connection diagram of authors. The size of the node indicates the number of articles published (larger nodes equate to more articles), and color shade of the node indicates the total connection strength (bluer shade equates with a greater total connection strength). The thickness of the brown connector between authors indicates the strength of the connection (thicker translates to a closer connection).

The author analysis showed that Guermazi A ranks first among all indicators. Guermazi et al. extensively studied not only the pathological mechanism of KOA but also the factors influencing KOA and the effect of different treatments on MRI results in patients with KOA (33–35). The number of publications and total citations for Cicuttini FM were low, but the central medium ranked second. Cicuttini et al. focused on the standardization of MRI scans in patients with OA (36–38). Although the rankings of Hunter DJ were lower than those of Guermazi A, they were stable, proving that Hunter DJ has a certain influence in this field. Hunter focused on the analysis of risk factors affecting the clinical symptoms of patients with KOA and disease prediction and judgment using MRI technology (39–41). The authors' network connections demonstrate the strength of cooperation between the authors. Figure 5 shows that the total connection strength between Guermazi and Roemer, Eckstein, and other authors is high, indicating that they not only have a high number of publications in this field, but also have willingness and strong ability to collaborate. The results show that many authors are involved in this field, and that the research content affects the degree of the authors' attention. Guermazi A is a leading author in this field.

### 3.6 Reference analysis

**Figure 6:**
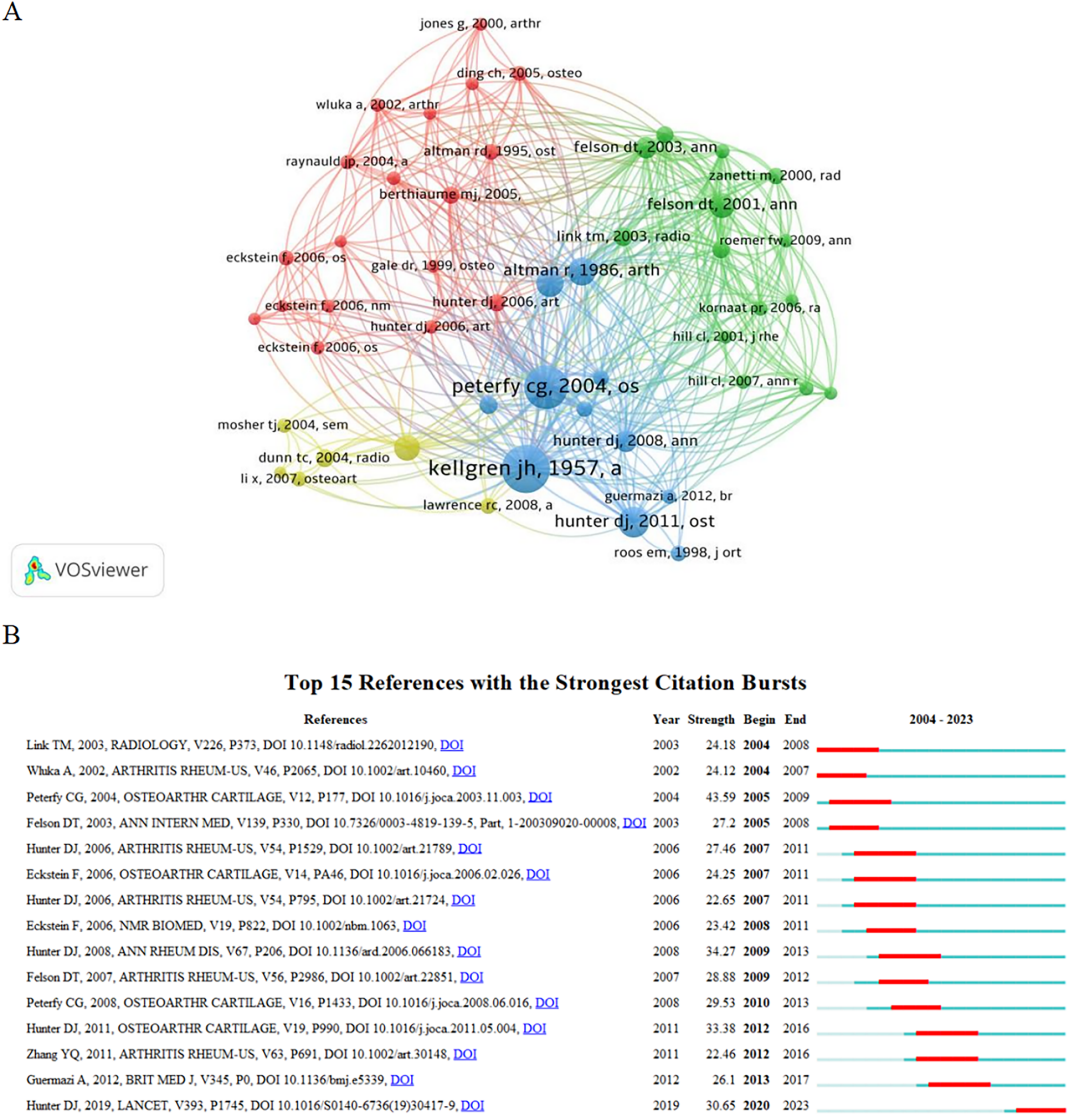
**(A)** Reference cocitation analysis diagram. The node size represents the number of cocitations, meaning that the larger the node, the more times the literature is cited. Different colors represent different species. The references of the same color have similar research content and are closely related. **(B)** Reference outbreak analysis diagram. “Strength” represents the burst intensity of the literature. The red line represents the concentrated outbreak time for this reference. The light blue line in the figure shows that although the literature is not a reference at this stage, it also obtains some citations.

According to the citation analysis, 43,288 references were cited in this study, and the top 10 were cited more than 177 times. The top three references with the highest total number of citations were the Radiological Assessment of Osteoarthrosis (42) (732), Whole-organ Magnetic Resonance Imaging Score (WORMS) of the knee in Osteoarthritis (43) (634), and Evolution of Semi-quantitative Whole-joint Assessment of Knee OA: MOAKS (MRI Osteoarthritis Knee Score) (44) (378) (Table 6). Figure 6A shows the total cited network connection strength of the references.

**Table 6:** Top 10 references of research on the application of MRI in KOA.

Rank	Co-citations	Cited reference	Journals	Authors (publication year)
1	732	Radiological assessment of osteo-arthrosis (42)	Annals of Rheumatic diseases	Kellgren JH (1957)
2	634	Whole-Organ Magnetic Resonance Imaging Score (WORMS) of the knee in osteoarthritis (43)	Osteoarthritis Cartilage	Peterfy CG (2004)
3	378	Evolution of semi-quantitative whole joint assessment of knee OA: MOAKS (MRI Osteoarthritis Knee Score) (44)	Osteoarthr Cartilage	Hunter DJ (2011)
4	332	Development of criteria for the classification and reporting of osteoarthritis. Classification of osteoarthritis of the knee. Diagnostic and Therapeutic Criteria Committee of the American Rheumatism Association (45)	Arthritis Rheum	Altman R (1986)
5	319	Validation study of WOMAC: a health status instrument for measuring clinically important patient relevant outcomes to antirheumatic drug therapy in patients with osteoarthritis of the hip or knee (46)	J Rheumatol	Bellamy N (1988)
6	288	The osteoarthritis initiative: report on the design rationale for the magnetic resonance imaging protocol for the knee (47)	Osteoarthr Cartilage	Peterfy CG (2008)
7	287	The association of bone marrow lesions with pain in knee osteoarthritis (48)	Ann Intern Med	Felson DT (2001)
8	230	The reliability of a new scoring system for knee osteoarthritis MRI and the validity of bone marrow lesion assessment: BLOKS (Boston Leeds Osteoarthritis Knee Score) (49)	Ann Rheum Dis	Hunter DJ (2008)
9	229	Bone marrow edema and its relation to progression of knee osteoarthritis (50)	Ann Intern Med	Felson DT (2003)
10	178	Osteoarthritis: MR imaging findings in different stages of disease and correlation with clinical findings (51)	Radiology	Link TM (2003)

Reference burst analysis showed that 15 references had a high burst intensity. Since 2011, literature focusing on the relationship between MRI and fluctuations in knee pain, bone marrow lesions, and changes in synovitis, as well as the diagnostic advantage of MRI for KOA, has received considerable attention (Figure 6B). According to the results, references with a large number of cocitations also had a high outbreak intensity. Their research content involved the diagnosis of KOA, research and development of evaluation tools, KOA risk factors, clinical stage, and other aspects that have attracted considerable attention.

### 3.7 Keyword analysis

#### 3.7.1 Keyword co-occurrence analysis

**Figure 7:**
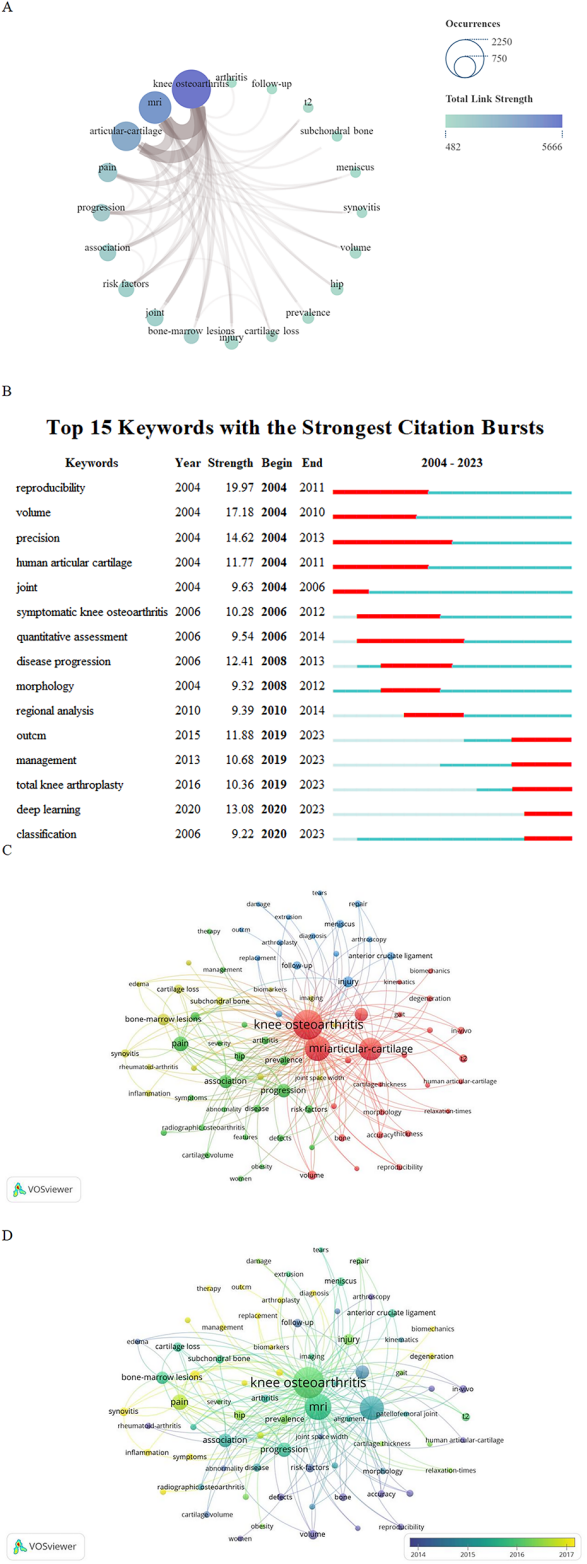
**(A)** Top 20 keyword network connection graph. The size of the node indicates the number of published articles related to the topic of the keyword (the larger the node, the higher the frequency of keyword co-occurrence, the more literature). The color shadow of the node indicates the total connection strength (the bluer the color, the greater the total connection strength, indicating that the more important the keyword is in the field). The brown joint thickness varies between keywords; the thicker the connector, the tighter the connection. The size of the node represents the number of articles published related to the topic of the keyword (the larger the node, the higher the keyword co-occurrence frequency, the more literature), and the shade of the node represents the total connection strength (the bluer the color, the greater the total connection strength, indicating the more important the keyword in the field). The thickness of the brown joint between the key words is different; The thicker the connector, the tighter the connection. **(B)** Explosive word analysis diagram. “Strength” represents the burst intensity of the literature. The red line represents the concentrated outbreak time of the keyword. The light blue line in the figure shows that although this keyword is not a reference at this stage, it is also cited. **(C)** Keywords cluster analysis. Different colors represent different clusters. The size of the node is related to occurrence; the larger nodes equate with greater occurrence. The color of the node represents the category to which the keywords belong, and the same color means that these keywords belong to the same cluster. **(D)** Key words network connection diagram. The node size represents the number of occurrences of the keyword. Larger node indicates more frequent occurrence of the keyword. The color of the node represents the keyword. Darker color indicates earlier appearance of keywords.

In total, 6,178 keywords appeared in this study, 93 of which appeared >50 times. “Knee osteoarthritis” (2,189) had the highest frequency, followed by “MRI” (1,463) and “articular cartilage” (1,180) (Table 7). The results of the keyword co-occurrence analysis showed that “KOA” and “MRI” were ranked as the top two keywords. “Articular and cartilage” was the third most common keyword, with 1,180 co-occurrences, indicating that the application of MRI in patients with KOA has focused more on the articular cartilage. The keyword connection strength network shows that MRI is applied to many aspects of KOA; however, it focuses on the study of knee cartilage and symptoms of KOA (Figure 7A). The evaluation and application of medical knee MRI in KOA cartilage, such as the volume and thickness of the cartilage, is the most important aspect in this field.

**Table 7:** Top 10 keywords in terms of co-occurrence frequency and mediation centrality in the field.

Rank	Occurrences	Keywords	Centrality	Keywords
1	2,189	Knee osteoarthritis	0.08	Anterior cruciate ligament
2	1,463	MRI	0.06	Knee osteoarthritis
3	1,180	Articular cartilage	0.05	*In vivo*
4	477	Pain	0.05	Accuracy
5	437	Progression	0.04	Thickness
6	406	Association	0.04	Radiographic osteoarthritis
7	349	Joint	0.04	Cartilage volume
8	233	Bone marrow lesions	0.04	Abnormality
9	231	Hip	0.04	Patellofemoral joint
10	199	Prevalence	0.03	Risk factor

#### 3.7.2 Analysis of outbreak-related words

Through the discovery of intermediary centrality, the “anterior cruciate ligament”, “cartilage thickness”, “cartilage volume”, and “patellofemoral joint” have attracted the attention of researchers. However, as the overall intermediary centrality was low, the reliability of the conclusions required additional data.

Combined with an analysis of explosive words, it was possible to further clarify the existing research hotspots. “Reproducibility” and “volume” were keywords with the highest outbreak intensities. It has been proven that before and after 2010, MRI of KOA focused on image reproduction and volume measurement, especially human articular cartilage. MRI of the knee joint also showed a high outbreak intensity in 2006–2012. As an artificial intelligence (AI) analysis method, “deep learning” experienced a high outbreak intensity in 2020. Therefore, in the next few years, the number of articles combining AI with KOA imaging is expected to increase (Figure 7B). Additionally, MRI may be used as a standardized method in clinical research on KOA and may promote the formulation of standards for imaging and symptomatology. From the analysis of the results, different research stages have different research hotspots, but compared to “management” and “classification,” “deep learning” has a high outbreak intensity after 2020, which will be a research hotspot in the next few years.

#### 3.7.3 Keyword cluster analysis

The keyword cluster analysis clearly indicated the widespread application of medical knee MRI in KOA risk factors, clinical treatment, and pathological injury research. Four cluster groups were formed by cluster analysis, and “KOA”, “pain”, “bone marrow lesions”, and “injury” were the main keywords in the four clusters (Figure 7C). According to the cluster analysis results, the relationship between the characteristics of pathological lesions, such as cartilage and subchondral bone injury, bone marrow defects, and inflammation, and KOA is the main component of MRI research. As shown in Figure 7D, the application of MR technology in KOA is extensive and involves risk factor analysis, pathological changes, diagnosis, and treatment.

## 4 Discussion

### 4.1 Methodological analysis of the bibliometrics

As the main software for this bibliometric analysis VOSviewer, CiteSpace, and SCImago Graphica played significant roles as the main software packages for this bibliometric analysis. VOS viewer is a free computer program, which was expanded by van Eck et al. (52), who provided a viewer with a variety of bibliometric maps, including density views and cluster density views. The VOSviewer view feature has a significant advantage for map presentation, with at least a moderate number of items (e.g., at least 100 items), while also containing the cocitation and co-occurrence analysis of various elements, including countries, institutions, and authors. The CiteSpace treatment of a bibliometric atlas is more complex. However, this approach also has unique advantages. For instance, it can not only delete duplicate literature in the data but also analyze the outbreak word for each element and then show the research hotspot in a certain field in different time periods. At the same time, unlike VOSviewer, CiteSpace calculates the mediation centrality of content in a certain element. Mediator centrality is the main indicator of bibliometrics, and nodes with high mediation centrality may lie between two large clusters or subnetworks. High mediator centrality makes it easier to gain attention in the field. In addition, CiteSpace can conduct an analysis of the double-graph superposition of journals and show the relationship between different categories of journals with color lines, which allows readers to understand the intermediary centrality of relevant content in a certain element. As a tool for visual operating systems that has been developed in recent years, SCImago Graphica does not require code and can transform various data sources (53). SCImago Graphica aims to combine high levels of expressive force with an easy-to-use interface. Moreover, the design of SCImago Graphica is highly flexible for the image presentation of the data and it also can realize visual communication of the data.

In addition, VOSviewer was employed to complete the data analysis of countries, institutions, journals, authors, references, publications, and keywords; obtain specific data of different elements; and display them in the form of an analytic knowledge graph. However, CiteSpace was used to conduct the mediation centrality of each element, superposition of journal dual graphs, and correlation analysis of outbreak words to compensate for the deficiencies of VOSviewer. SCImago Graphica was used to create a national geographic distribution map and a network connection map between the author and keywords so that the total connection strength between the author and keywords was more directly displayed.

Some previous bibliometric analyses have combined VOSviewer, CiteSpace, and SCImago Graphica. For example, Wang et al. analyzed the top ten0 articles in the field of chronic fatigue syndrome by combining the three packages, and all three were used to map knowledge. These results appear to be more intuitive (54). A similar bibliometric analysis of Titis obliterans was performed by Liu et al., who used VOSviewer to analyze academic collaborations between different countries, institutions, and authors, while simultaneously drawing a visual map of the cocited authors, journals, references, and co-occurrence keywords. CiteSpace was also used to supplement the periodic double-image superposition and outburst word analysis. The Scimago Graphica application was used to map academic cooperation between different countries/regions, similar to the method used in the present study (55). However, in their study, the mediation center results were not displayed, and the in-depth discussion of the results in the Discussion was reduced, with only a brief explanation of the results. The filterometric analysis of the rough set was similar to that presented by Yu et al., where SCIE and SSCI were selected as literature sources and article types (articles or comments). Moreover, a network analysis of countries, regions, and authors was introduced, and research hotspots and trends were explored through the cocitation of keywords and analysis of outbreak-related words (56). However, in contrast to their research, our research focuses on the cocitation analysis of different elements and the top ten contents of the elements, which helps readers grasp the main content and hot spots in the analysis of each element and then helps scholars find the direction of in-depth development. Later, they conducted a quantitative bibliometric analysis of the publications of Applied Intelligent Magazines, where they divided content into different time phases, focusing on the time trend and proportion of factors in the number of applied intelligence articles and citations (57). However, relatively few studies have analyzed these results.

Compared to similar bibliometric analyses, the present study focuses on the number of research elements, cocitation or cocitation number of times and mediation centrality in the process of analysis. The display of the knowledge graph does not pay attention to the comparison of the results of the three drawing software programs but focuses on the three software programs to complement each other and show the research results in a more comprehensive way. A very large space is used to conduct in-depth discussions of the results and analyze future research trends and hotspots.

### 4.2 Global publication status and quality analysis of research in the application of medical knee MRI in KOA

This study included 2,904 studies. Based on the annual publication trend, research on medical knee MRI in KOA is increasing. The United States has published many studies in this field. Moreover, its intermediary centrality and total citation volume are at the forefront, have a certain output in this field, and have great influence. Several factors influenced the outcome. First, the progress in MRI technology in the United States cannot be ignored. In the functional evaluation of real-time MRI, American scholars have analyzed the morphological characteristics of the cartilage by studying its volume and thickness. Simultaneously, the automatic segmentation of the cartilage and meniscus morphology was analyzed based on a convolutional neural network (CNN), which has a significant influence in this field (58). In studies on the integrated management of KOA, studies from the United States had high numbers of citations (59). European countries, such as Germany and France, also have a large output in this field and strong connections with the United States. In authors from these countries co-operation with the United States, they not only elaborated on the current situation of the application of medical knee joint MRI in KOA but also discussed the related MRI technology and analyzed the application characteristics of MRI technology in KOA (60). At the same time, they established a magnetic resonance observation cartilage repair tissue score using the MRI semi-quantitative scoring method, which can evaluate the extent of joint surface damage by scoring the volume of the cartilage defect, degree of integration with adjacent cartilage, surface and structure of the repaired tissue, and signal strength of the repaired tissue on PD-weighted images (61). These studies further promotes the progress of medical knee joint MRI technology and helps to improve the accuracy of diagnosis. These advances in MRI technology are evident in these influential countries.

China has a large number of publications and citations, which is related to its increasing medical investments in recent years. Indeed, owing to the convenience of MRI examinations and the increased incidence of KOA in patients, Chinese scholars have been paying more attention to the application of MRI technology in KOA in recent years. By integrating meniscal and femorotibial radiomic features to predict radiographic KOA incidence using neural networks, Li et al. has also suggested that the predictive value of JS-RM, integrating meniscus and cartilage features, improves the incidence of imaging KOA, which contributes to the early diagnosis of KOA (62). YeQ et al. used MRI to analyze the subpatellar fat pad in patients with KOA, which further supports that imaging omics changes of the subpatellar fat pad were associated with the severity of KOA and knee structural abnormalities in the elderly (63). Analysis of KOA risk factors using MRI can help to evaluate and screen patients with early KOA and provide early intervention in patients with microscopic injury to improve patient outcomes. However, Chinese scholars have focused more on analyzing the risk factors of KOA using MRI, while little research has been conducted on the technological progress of MRI applications in KOA, which may explain the lower total number of citations of Chinese articles. Therefore, while studying MRI detection of KOA, strengthening technical cooperation with scholars in the United States and European countries may further improve the output.

In the analysis of issuing institutions and journals, the results demonstrate the value of MRI in interdisciplinary applications for patients with KOA. Boston University ranked first in terms of the number of published articles and citations, suggesting that it has an important influence in this field. The University of Tasmania and University of Iowa have a large number of published papers, but their total citations and intermediary centralities are low, suggesting that they must work closely with other core institutions to improve their research quality. Many indicators of the University of Sydney are in the top ten, proving that the institution has certain achievements in this field, with a focus on biomechanical and clinical research related to KOA, demonstrating MRI is valuable for the development of interdisciplinary KOA research (64–66).

Journal analysis showed that Osteoarthritis and Cartilage was the most important journal for MRI applications in KOA. Combined with a high IF, it can serve as a core journal in a field that scholars can follow to advance their research. As early as 1994, Osteoarthritis and Cartilage published articles on the use of MRI techniques to assess the extent of cartilage damage in patients with KOA and the clinical advantages of related interventions (67, 68). Later, the “Whole-organ magnetic resonance imaging score (WORMS) of the knee in osteoarthritis” (43), published in the journal, received very high citations. This further promotes the application of MRI in the clinical diagnosis of KOA. In recent years, studies on semiquantitative techniques for medical knee MRI in KOA, MRI characteristics of KOA biochemical components, and risk factors for KOA have been published in this journal (69–71). As a journal in this field, research published in KOA has made up for the lack of research on MRI image characteristics, risk factors, and formulation of imaging diagnostic criteria, providing a certain reference for scholars.

Although Annals of Rheumatic Diseases has a small number of publications, the total number of citations and co-cited are both high, indicating that the quality of the articles in this field is high, and the content has attracted the attention of many scholars. The “radiological assessment of osteo-arthrosis”, published in 1957, remains the main radiological grading standard for KOA. Arthritis & Rheumatology, Arthritis Care & Research, BMC Musculoskeletal Disorders, and other journals had relatively late publication times, suggesting that MRI in KOA has applications in the fields of nursing and rheumatology (Figure 4A).

Based on an analysis of the authors and references, MRI has certain applications in exploring the pathogenesis of KOA and standardizing research. Although the number of publications involving research on medical knee MRI in KOA has increased annually, references with strong outbreak intensities were mostly published before 2010 (Figure 6B). Therefore, scholars need to thoroughly investigate the academic research space based on previous studies of MRI in KOA to improve their influence.

The number of publications, total citations, and intermediary centrality of Guermazi A in this field are relatively high, which is sufficient to demonstrate the significant influence of the author in this field. Indeed, his 2023 review further highlights the transition of KOA from cartilage “wear” to whole-organ disease from an MRI technology perspective. Simultaneously, the advantages of semi-quantitative, quantitative, and component MRI in patellofemoral joint evaluation were analyzed (13). This provides more rational advice for driving the development of diagnostic criteria for imaging of KOA types such as patellofemoral arthritis.

Based on an analysis of the authors and references, MRI has certain applications in exploring the pathogenesis of KOA and standardizing research. Although the number of publications involving research on medical knee MRI in KOA has increased annually, references with strong outbreak intensities were mostly published before 2010 (Figure 6B). Therefore, scholars need to thoroughly investigate the academic research space based on previous studies of MRI in KOA to improve their influence.

### 4.3 Trend analysis of the application of medical knee MRI in KOA

From the perspective of keyword intermediary centrality, “articular cartilage”, “cartilage volume”, “anterior cruciate ligand”, “patellofemoral joint”, and “thickness” have received increasing attention in previous MRI studies on KOA (Figure 5). KOA is characterized by cartilage volume loss and multifactorial pain. Morphomorphology-based MRI techniques help determine the relationship between injury and pain in cartilage volume. In 2017, Schaefer et al. used self-developed local and regional cartilage segmentation (LACS) software to analyze the relationship between cartilage volume and pain. Their validity and sensitivity measurements of the medial femoral cartilage volume in the central load-bearing area demonstrated that LACS was fast, responsive, and associated with radiological and pain progression (72). Other researchers used a 3D CNN to measure the knee cartilage on MRI and used the volume measurement results provided by clinical experts as a reference. They found that the accuracy of measurement based on 3D-CNN was similar to the data provided by experts, which is of certain value for simplifying the operation process of MRI and improving the accuracy of cartilage volume measurement (73, 74). The semi-quantitative determination of cartilage by Tack A et al. using KneeCaP software also indicates the reliability of this technique (75). However, we found that MRI technology was used to measure the volume of KOA cartilage and in other tissue measurements of the knee joint (e.g., meniscus, subpatellar fat pad, and bone marrow lesions) (63, 76, 77). The measurement of these sites and injuries can not only help to clarify the risk factors of KOA but can also be of great value to guide the treatment of KOA. For example, 2 years before the diagnosis of KOA, changes in the subpatellar fat pad signal and effusion-synovitis volume predict the accelerated development of KOA. The odds of signal change in patients with possible KOA significantly increased 1 year before the diagnosis of KOA. When this feature is defined, early MRI examination and prevention of the relevant population are crucial for delaying the onset. In addition, the measurement of thigh adipose tissue by MRI helps to clarify pain in patients with KOA and provides a reference to delay the deterioration of pain symptoms in patients (78, 79).

The relationship among pain, bone marrow lesions, and knee MRI findings has been increasingly investigated. However, from the perspective of “in vivo”, “disease progression”, “hip”, “joint”, and other keywords, MRI is more commonly applied in clinical research of KOA. One contributing factor may be the lack of adequate experimental animal studies in this field, which may be due to the limited availability of animals (80–82). Total knee replacement was a popular topic that emerged in 2016, while the application of MRI in the treatment of advanced KOA has also been studied (83).

Since 2020, “deep learning” has had the highest burst intensity among all keywords, indicating that it has received significant attention in medical knee MRI research on KOA. Deep learning is a machine-learning method that has produced the most advanced results in many classical computer vision and image analysis problems (84, 85). Convolutional neural networks (CNNS) and recurrent neural networks (RNNS) in deep learning have been developed for use in various aspects of medicine (86). NamS et al. found that deep learning is more represented in cumulative neural networks applied in radiology and medical imaging. Compared to RNN, models using images for classification or prediction tasks are mainly based on CNN, the majority of which are ResNet and GoogLeNet (87). This is also one of the reasons why CNN are widely used in medical knee joint MRI.

In medical knee MRI studies, deep learning is widely used to evaluate and segment knee cartilage injuries. This method is advantageous in terms of accuracy and time (88, 89). MRI measurement of KOA risk factors helps assess patient severity and advance prevention and treatment. Zhao et al. applied a CNN to evaluate the MRI characteristics of patients with KOA and found that knee effusion/synovitis and cartilage loss were the most common abnormal manifestations associated with pain severity (90). However, in clinical applications, MRI is not as accurate as color Doppler flow imaging (CDFI) for fluid measurement. Therefore, combining MRI detection results based on deep learning with CDFI and the clinical symptoms of patients and extracting clinical features of MRI while quantifying effusion can help further understand the MRI features of patients with KOA injury and guide treatment. Simultaneously, the application of deep learning in medical knee MRI makes the detection and accurate diagnosis of KOA risk factors more efficient (91, 92). Although current MRI diagnostic criteria for KOA have not been developed, the application of deep learning in MRI will be very helpful in promoting research in this field.

We also noted that MRI of the medial knee joint is not only focused on articular cartilage, subpatellar fat pad, and bone marrow edema in KOA. Subpatellar fat pad lesions and bone marrow edema are associated with the onset and clinical symptoms of KOA; however, deep learning has only a few applications in this field (16, 93–95). Therefore, we believe that the application of deep learning should not only focus on the segmentation and injury evaluation of KOA cartilage but also place more emphasis on the measurement of bone marrow lesions, subpatellar fat pad signal abnormalities, cruciate ligament lesions, and meniscal injury and their value in the diagnosis of KOA. This will help to promote the establishment of MRI diagnostic criteria for KOA and help to evaluate the severe staging of KOA and guide clinical intervention.

Therefore, deep learning technology will be applied to the evaluation of cartilage damage and the prediction of risk factors and to the imaging relationship between other knee tissue injuries and KOA and promote the development of MRI diagnostic criteria for KOA. The use of CNN will help improve the efficiency of medical knee-joint MRI technology in KOA applications. Owing to the high burst intensity of deep learning and its practical value, deep learning will be widely used in MRI applications in the future, and the number of related studies will continue to increase.

Keyword-clustering analysis reveals the clustering and collaborative relationships of keywords in a more intuitive manner. In the “pain” cluster, “progression”, “hip”, and “prevalence” were the main keywords, suggesting that the study of pain and multiple joints has a certain value and that pain affects the incidence of KOA. For example, Girdwood et al. assessed the cartilage health of the patellofemoral and tibiofemoral CIA compartments using a semiquantitative MRI osteoarthritis score. They also found that hip rotation strength may be associated with the deterioration of function, exacerbation of symptoms, and cartilage health after ACLR (96).

As the main symptom of KOA, the relationship between cartilage damage and pain is clear (97, 98). Due to the good performance of MRI in evaluating cartilage damage, its application in different interventions for knee cartilage repair has attracted attention. Various interventions, including autologous chondrocyte implantation, mesenchymal stem cell therapy, and acupuncture, use MRI to detect their effects on cartilage repair. However, high-quality meta-analyses have little application in this regard. Therefore, further use of MRI to evaluate the relief of cartilage repair and KOA pain can provide high-level clinical evidence and improve the clinical treatment of KOA.

Through “injury” clustering, we found that the combination of MRI of the affected knee joint and anterior cruciate ligament has been extensively studied. However, Whittaker et al. found that semiquantitative syntheses identified moderate-certainty evidence that cruciate ligament, collateral ligament, meniscal, chondral, patellar/tibiofemoral dislocation, fracture, and multistructural injuries increased the odds of symptomatic OA (99). Despite this, it is difficult to further quantify these injuries from the imaging results and establish the relationship between KOA disease progression, rehabilitation, and other treatments. This is especially true before a clear diagnosis of KOA can be made without the help of radiography. At the same time, there are few studies on whether the treatment results of these injuries can be quantified and whether the timespan of clinical efficacy is different. Further exploration of the applications of CNN in this field is required. Therefore, while MRI is applied to KOA, attention needs to be paid not only to the cartilage but also to the multistructural damage of the knee. Understanding the characteristics of different injuries and conducting quantitative analysis using MRI not only contributes to the establishment of imaging diagnostic criteria for KOA but also provides data support for subsequent treatment. The relationship between synovitis and KOA has also gained attention as a keyword that has appeared frequently in recent years. Fan et al. found that a higher baseline score in patients with KOA with effusion synovitis of osteophytes detected by MRI in almost all compartments was significantly associated with increased total knee pain, weight-bearing pain, stiffness, and physical dysfunction after adjusting for age, sex, and body mass index, suggesting that fluid accumulation is also one of the factors for the symptoms of KOA (100). Overall, cluster analysis of keywords revealed that MRI technology has been applied to many aspects of KOA research, promoting progress in the study of KOA etiology, pathology, and clinical therapeutics.

To date, our study found that medical knee MRI is widely involved. Among these, morphometric measurements and cartilage evaluations are the most widely used. MRI can also be used to measure the knee ligament, meniscus, subpatellar fat pad, and smooth capsule fluid. However, there are no effective quantification standards for image segmentation, guidance prevention, or clinical intervention. Further research is needed to measure and quantify knee microinjuries using MRI for the diagnosis of KOA of different severities. In the current study, the measurement of MRI still required the use of radiograph, CDFI, and expert evaluation methods to evaluate its effectiveness. The emergence of deep learning may drive advances in MRI in all aspects of KOA applications.

### 4.4 Advantages and limitations

The focus of this study was to clarify the status quo of the application of medical knee MRI in KOA through bibliometric technology, and to explore the outline and hotspots of the future development of this field by analyzing the key content to provide a reference for readers to clarify the main content and direction of this field. However, this study had some limitations. Non-English publications were excluded, resulting in language bias. In addition, studies published before 2004 were excluded, and high-quality articles published in 2024 may not have been prominent because of their short study duration. At the same time, we did not include a literature search on central brain imaging of patients with KOA. In the future, we will consider including research in this field.

## 5 Conclusion

This study introduced the global status and trends in the use of medical knee MRI in patients with KOA. Large-scale studies have been conducted in this regard. Content in this field can be published in orthopedics, rheumatology, sports medicine, rehabilitation medicine, and many other journals. Applications of medical knee MRI technology in KOA treatment include the following: (1). MRI was used to assess the relationship between the characteristics of local tissue damage, pathological changes, and clinical symptoms. (2). The risk factors of KOA were analyzed by MRI to determine the early diagnosis of KOA. (3). MRI was used to evaluate the efficacy of multiple interventions for KOA tissue damage (e.g., cartilage defects, bone marrow edema, bone marrow microfracture, and subchondral bone remodeling). Artificial intelligence, particularly deep learning, will become the focus of MRI applications in KOA and this topic will attract increasing attention from scholars. At the same time, deep learning technology will be applied to the evaluation of cartilage damage and the prediction of risk factors and to the imaging relationship between the injury of other knee tissues and KOA and will promote research on MRI diagnostic criteria for KOA.

## Data Availability

The original contributions presented in the study are included in the article/Supplementary Material, further inquiries can be directed to the corresponding author.
